# The effect of a therapeutic regimen of Traditional Chinese Medicine rehabilitation for post-stroke cognitive impairment: study protocol for a randomized controlled trial

**DOI:** 10.1186/s13063-015-0795-x

**Published:** 2015-06-16

**Authors:** Jia Huang, Zhengkun Lin, Qin Wang, Feiwen Liu, Jiao Liu, Yunhua Fang, Shanjia Chen, Xiaoxuan Zhou, Wenjun Hong, Jinsong Wu, Natalia Madrigal-Mora, Guohua Zheng, Shanli Yang, Jing Tao, Lidian Chen

**Affiliations:** College of Rehabilitation Medicine, Fujian University of Traditional Chinese Medicine, No. 1 Huatuo Road Shangjie Minhou, Fuzhou, 350122 China; Fujian Key Laboratory of Exercise Rehabilitation, No. 282 WUSI Road, Gulou, Fuzhou, 350003 China; National Rehabilitation Research Center of Traditional Chinese Medicine, No. 1 Huatuo Road Shangjie Minhou, Fuzhou, 350122 China; Friedrich-Alexander University Erlangen-Nuremberg, Schlossplatz 4, 91054 Erlangen, Germany; Fujian University of Traditional Chinese Medicine Subsidiary Rehabilitation Hospital, No. 282 WUSI Road, Gulou, Fuzhou, 350003 China; Fujian University of Traditional Chinese Medicine, No. 1 Huatuo Road Shangjie Minhou, Fuzhou, 350122 China

**Keywords:** Stroke, Cognitive impairment, Acupuncture, Cognitive training, Randomized controlled trial

## Abstract

**Background:**

Post-stroke cognitive impairment (PSCI) lessens quality of life, restricts the rehabilitation of stroke, and increases the social and economic burden stroke imposes on patients and their families. Therefore effective treatment is of paramount importance. However, the treatment of PSCI is very limited. The primary aim of this protocol is to propose a lower cost and more effective therapy, and to confirm the long-term effectiveness of a therapeutic regimen of Traditional Chinese Medicine (TCM) rehabilitation for PSCI.

**Methods/Design:**

A prospective, multicenter, large sample, randomized controlled trial will be conducted. A total of 416 eligible patients will be recruited from seven inpatient and outpatient stroke rehabilitation units and randomly allocated into a therapeutic regimen of TCM rehabilitation group or cognitive training (CT) control group. The intervention period of both groups will last 12 weeks (30 minutes per day, five days per week). Primary and secondary outcomes will be measured at baseline, 12 weeks (at the end of the intervention), and 36 weeks (after the 24-week follow-up period).

**Discussion:**

This protocol presents an objective design of a multicenter, large sample, randomized controlled trial that aims to put forward a lower cost and more effective therapy, and confirm the long-term effectiveness of a therapeutic regimen of TCM rehabilitation for PSCI through subjective and objective assessments, as well as highlight its economic advantages.

**Trial registration:**

This trial was registered with the Chinese Clinical Trial Registry (identifier: ChiCTR-TRC-14004872) on 23 June 2014.

**Electronic supplementary material:**

The online version of this article (doi:10.1186/s13063-015-0795-x) contains supplementary material, which is available to authorized users.

## Background

Cognitive impairment is considered as one of the major long-term effects after a stroke [1, 2]. In China, post-stroke cognitive impairment (PSCI) occurs in 55.9 % of all patients who suffer a stroke [3]. In the United States, the total annual costs of stroke are thought to increase to US$240.67 billion by 2030, and a large part of these costs is caused by cognitive impairment [4]. In addition to the direct effect on the quality of life of patients and their families [5], there are also indirect effects of this cognitive dysfunction on functional recovery, due to the inability to actively participate in functional training and the failure to follow task instructions [6]. Cognitive impairment can influence patients’ self-confidence and may cause depression, anxiety, and unwillingness to take part in daily activities [7]. The presence of cognitive impairment can also be an important predictor of recovery and has been associated with the risk of recurrent stroke [8]. Therefore, interventions for PSCI are of paramount importance. Unfortunately, the effective interventions of PSCI are very limited [9]. There are some effective drug therapies, such as cholinesterase inhibitors (for example donepezil [10]), cerebral circulation improvement agents, antidepressants, and other investigational drugs; however they have unavoidable side effects, such as anorexia, nausea, vomiting, diarrhea, fatigue, and vertigo [11]. New therapies, such as music therapy [12] and hyperbaric oxygen therapy, have few evidence-based medical studies and more research is needed to determine their safety and efficacy [13].

Cognitive training (CT), which typically involves guided practice on non-specific cognitive activities and specific cognitive functions [14], is used worldwide [15]. There is substantial evidence on the use of CT for cognitive impairment, but systematic reviews suggest that current CT for PSCI still has limited effectiveness [16], and patients cannot improve their cognitive function quickly enough to return to daily life. Therefore, new treatments or comprehensive therapeutic strategies should be of utmost importance in future studies in order to avoid the previously mentioned limitations [17].

Acupuncture is a complementary and alternative therapy. It has a long history in the management of patients with neurological symptoms [18], which has revealed promising cost-effectiveness results [19]. Many studies have highlighted the potential positive effects of acupuncture as a promising treatment for patients with PSCI [20]. Promoting cholinergic neural transmission, facilitating dopaminergic synaptic transmission, enhancing neurotrophin signaling, suppressing oxidative stress, and attenuating apoptosis are some of the proposed mechanisms by which acupuncture improves cognitive function [21]. However, the efficacy of acupuncture is yet to be unequivocally defined. Since the acupoint selection is heterogenous between studies, the conclusions drawn are limited. Thus, the effect of appropriate acupoints needs further research. Only two acupoints, Baihui (DU20) and Shenting (DU24), will be used in this therapeutic protocol, based on our previous study for PSCI treatment [22, 23].

Currently, no single acupuncture intervention has proved to be unequivocally beneficial for PSCI recovery [20]. Systematic reviews have proposed that acupuncture in combination with other therapies can improve cognitive functions significantly [24]. Evidence also suggests that CT combined with other interventions enhances treatment benefits [14] since acupuncture focuses on general cognitive function, while CT addresses one specific impaired cognitive aspect [25]. Their combination, also considered a therapeutic regimen of Traditional Chinese Medicine (TCM) rehabilitation, may prove better at improving cognitive function after stroke. This protocol is sponsored by the 12th Five-year Plan project of the Ministry of Science and Technology of the People’s Republic of China (grant number: 2013BAI10B01).

Further evidence and recommendations for clinical practice supporting a therapeutic regimen of TCM rehabilitation are currently insufficient. Although many studies have addressed the potential use of acupuncture to improve cognitive function, few have explored the effects of a therapeutic regimen of TCM rehabilitation in patients with PSCI [26, 27]. Because of limitations in design or sample size, some of these studies provided equivocal results, thus enhancing the need to conduct high methodological quality trials with large samples and longer follow-up periods. The purpose of this randomized controlled trial is to test the cost-effectiveness of TCM rehabilitation therapy and confirm its effectiveness for PSCI treatment.

## Methods/Design

### Study design

**Fig. 1:**
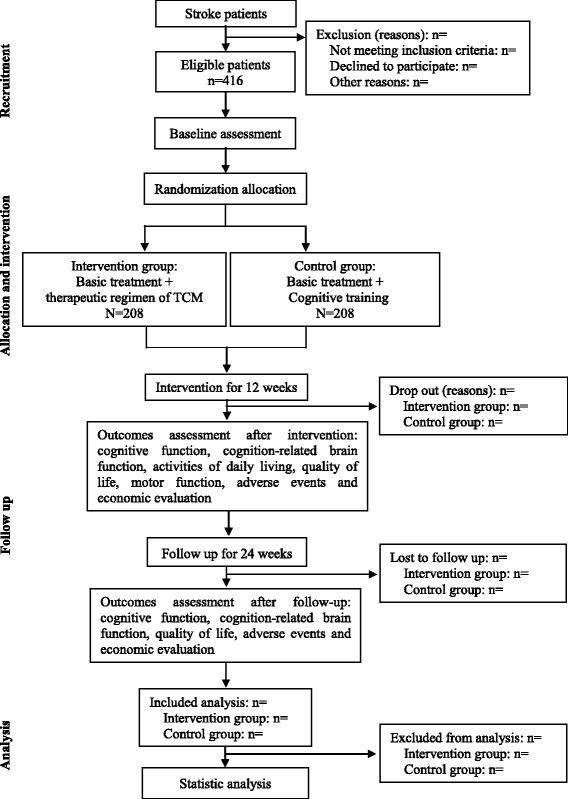
CONSORT flow diagram of study design

The clinical study is a prospective, multicenter, large sample, randomized controlled trial designed with single-blinded assessments, which is being carried out in seven inpatient and outpatient stroke rehabilitation units. With prior written informed consent, a total of 416 eligible participants will be randomly allocated into the intervention group or control group in a 1:1 ratio. The control group will receive conventional treatment and CT, while, the interventional group will receive conventional treatment and a therapeutic regimen of TCM rehabilitation (CT + acupuncture). A 12-week intervention and 24-week follow-up period will be conducted for this trial. The relative outcomes will be measured at baseline, at the end of intervention (12 weeks), and after the 24-week follow-up period (36 weeks). Fujian University of Traditional Chinese Medicine (FJTCM), as a major undertaker of the study, is responsible for training rehabilitation therapists on standard operation procedure and supervising the progress of this trial in all clinical sites. In addition, the randomization and blinding will be performed by an independent statistician from the Center of Evidence Based Medicine, FJTCM. A flow diagram of this trial is presented in Fig. 1.

### Participants and recruitment

The participants will be recruited from seven inpatient and outpatient stroke rehabilitation units from FJTCM Subsidiary Rehabilitation Hospital, Guangdong Province Traditional Chinese Medical Hospital, The First Affiliated Hospital of Jinan University, Traditional Chinese Medicine Hospital of Xinjiang Uygur Autonomous Region, The Affiliated Huashan Hospital of Fudan University, Zhongshan Hospital of Traditional Chinese Medicine, and ShiyanTaihe Hospital, all in China. Our study will be advertised on each hospital’s internet homepages. Posters informing patients about this trial are displayed in several community rehabilitation centers and hospitals. Interested patients can contact the project leader (PL) through their therapists, via phone, email, or mail. After obtaining informed consent, the prospective participants are screened according to the inclusion and exclusion criteria. Those who comply with all criteria are eligible to participate. The eligible individuals are invited to participate in the trial and are directed to a diagnostic assessment by a neurologist, followed by rehabilitation assessments. Data on the design of the study is shown in Table 1.

**Table 1:** Study design

Items		Before intervention		Intervention period		Follow-up period	
		Screening	Evaluate^a^	Weeks 1-12	Evaluate^b^	Weeks 13-36	Evaluate^c^
Enrollment		×					
Informed consent		×					
Basic information		×					
Inclusion and exclusion criteria		×					
Randomization and allocation		×					
Primary outcomes	MMSE	×	×		×		×
Secondary outcomes	MoCA		×		×		×
	TAP		×		×		
	WMS		×		×		
	TMT		×		×		
	Stroop		×		×		
	MBI		×		×		×
	SF-36		×		×		×
	FMA		×		×		
	*f*MRI		×		×		
	ERP		×		×		
Treatment recorded				×		×	
Adverse events recorded				×		×	
Rehabilitation expenses questionnaire				×		×	×

^a^Evaluation in the week before intervention. ^b^Evaluation in the week after intervention. ^c^Evaluation in the week after follow-up. Abbreviations: MMSE, Mini-Mental State Examination; MoCA, Montreal Cognitive Assessment; TAP, Test of attentional performance; WMS, Wechsler memory scale; TMT, Trail Making Test A and B; Stroop, Stroop Test; MBI, Modified Barthel Index; SF-36, 36-Item Short Form Health Survey; FMA, Fugl-Meyer Motor Assessment; *f*MRI, Functional magnetic resonance imaging; ERP, Event-related potentials

### Inclusion criteria

This trial’s participants should meet all the following criteria: (1) Signed informed consent by the patient or their guardian to participate in this trial; (2) Present stroke diagnosis according to the fourth national academic conference on cerebrovascular disease’s Diagnostic Criteria for All Kinds of Cerebrovascular Disease [28], and confirmed by computed tomography or magnetic resonance imaging; (3) Mini-Mental Status Examination (MMSE) score ≤24; (4) First ever stroke and within one year post-stroke; (5) Aged between 45 to 75 years; and (6) Patient should be conscious, with stable vital signs.

### Exclusion criteria

Criteria for exclusion are as follows: (1) Patients who suffer from brain tumor, brain trauma, brain parasite disease, or other diseases that could cause cognitive impairment; (2) Patients with severe language, vision, and/or auditory impairment, or neuropsychiatric disorder precluding cognitive examination; (3) Patients who suffer from diseases related to cognitive impairment confirmed by anamnesis, doctors, or families, or with history of cognitive drug use before stroke; (4) Beck Depression Scale score >13; (5) Patients with alcohol or drug abuse; (6) Patients with severe illnesses, such as heart, liver, kidney, and endocrine diseases, and hematopoietic system disease; (7) Pregnant woman or breastfeeding women; or (8) Patients who are included in other clinical trials that would affect the evaluation results of this study.

### Sample size

The sample size calculation is based on the improvement of MMSE scores, which is used as the primary effect indicators to estimate. According to the similar published articles [29], the mean difference in MMSE scores before and after treatment is three points, with a standard deviation of 1.5. According to our pilot study, a therapeutic regimen of TCM rehabilitation (CT + acupuncture) can probably improve the MMSE by 3.52 points on average compared to the control group. Improvement will be measured according to the same size of the estimation formula:

1 $$ \mathrm{n}=2\left[{\left({u}_{\alpha }+{u}_{\beta}\right)}^2{\sigma}^2\right]/{\delta}^2 $$

With a type I error of 5 % (α = 0.05) and 90 % power (β = 0.10), it is estimated that a sample size of 173 participants per group will be required. Considering a 20 % dropout rate during the study, a minimum of 416 total participants is needed to reach the target 208 participants per group.

### Randomization and allocation concealment

Stratified-block randomization would be applied into the study, which will take each center as a stratification factor. Each eligible participant will be randomly assigned to either the intervention group or control group according to 1:1 equal proportion rule. The sequence of random allocation will be generated using the PLAN sentences of the statistical software SAS 9.3 (SAS institute Inc., Cary, NC, USA), by an independent statistician who will not be involved in this trial from the Center of Evidence Based Medicine, FJTCM. Also, the sequence of random allocation will be concealed to outcome assessors and the statistical analyst. Then, the eligible participants will be informed of their allocation result by the project manager via telephone after their baseline information has been assessed, and the therapists (including acupuncturist and cognitive therapists) will be notified of the allocation and arrange the allocated treatment directly for the patient.

### Blinding

In this trial, both the outcome assessors and the statistical analyst will be blinded to the randomization allocation and will not be involved in performance of the interventions. It will be, nevertheless, impossible to blind the participants, acupuncturists, and cognitive therapists since they will perform the interventional protocols. In the meantime, a specified project manager will be assigned to be in charge of the random allocated sequence and blind code of allocation, in which the intervention group or control group will be replaced by the letter A or B. Random parameters would be kept in the blind codes, using sealed opaque envelopes to conceal random allocation. Unblinding will only occur once all the outcome data collection is completed at 36 weeks.

### Ethical issues

This protocol has been approved by the Ethics Committee of FJTCM Subsidiary Rehabilitation Hospital (approval number: 2013KY-005-01), Guangdong Province Traditional Chinese Medical Hospital (approval number: B2014-020-01), the First Affiliated Hospital of Jinan university (approval number: (2014) Ethical review approval documents 25th), Traditional Chinese Medicine Hospital of Xinjiang Uygur Autonomous Region (approval number: 2013XE005), Affiliated Huashan Hospital of Fudan University (approval number: (2014) Interim review 262th), Zhongshan Hospital of Traditional Chinese Medicine (approval number: 2014ZSZY-LLK-002), and ShiyanTaihe Hospital (approval number: 2014001 Research review meeting 2nd). Each participating center will receive approval from the local institutional review board. Prior to study, signed informed consent will be obtained from each participant. The names of seven ethical bodies are listed in Additional File 1.

### Baseline tests

Descriptive data will be collected before randomization. The descriptive data includes gender, age, height, weight, nationality, education level, employment status, clinical syndrome (left or right hemiplegia), and the type, frequency, and location of the stroke at diagnosis. The National Institute of Health Stroke Scale, an 11-item evaluation tool to assess stroke severity, will be applied at baseline to determine whether the two groups are consistent before the intervention [30]. All subjects will be assessed with the following measures: the severity of cognitive impairment will be assessed using the Mini-Mental Status Examination (MMSE), as well as the Montreal Cognitive Assessment (MoCA); attention function will be measured with the Test of Attentional Performance (TAP); memory function will be tested with the Wechsler Memory Scale-Revised in Chinese (WMS-RC); executive function will be evaluated with the Stroop test, as well as the Trail Making Test; motor function will be measured with the Fugl-Meyer Motor Assessment Scores; activities of daily living (ADL) will be assessed with the Modified Barthel Index (MBI); and quality of life will be evaluated with the 36-Item Short Form Health Survey (SF-36). Moreover, the functional condition of the brain is examined by using a resting-state functional magnetic resonance imaging (fMRI) and event-related potentials (ERP).

### Intervention

The interventional group will receive a therapeutic regimen of TCM rehabilitation and conventional treatment, while the control group will only receive CT and conventional treatment. The conventional treatment consists of health education, conventional medical therapy, and routine rehabilitation training. The treatments will be performed five times per week, over 12 weeks. For quality management, all procedures performed by the healthcare staff participating in this study will be standardized, including in the study protocol, treatment methods, and assessments.

### Basic treatment

All participant will receive basic treatments following the Chinese Medical Association’s *Guidelines for the Prevention and Treatment of Cerebrovascular Disease in China (2007)* [31] and *Guidelines for Chinese stroke rehabilitation* [32]*.* Based on these guidelines and the patient’s condition, the treating physician will manage each case, including the use of drugs, stroke risk factor prevention, and rehabilitation therapies, as well as post-stroke comorbidity management including anticoagulant therapy, blood pressure, anti-platelet aggregation, heart disease, blood sugar, and lipid management.

### Health education

All the participants will receive an informational brochure with basic knowledge on stroke, based on the *Out of the Misunderstanding of Stroke Patients*’ *Rehabilitation* (the bestseller books for health education after stroke from the Chinese Association of TCM) [33]. In addition, a health lecture is offered to participants in the hospitals, covering various topics on stroke rehabilitation, including: the definition of stroke; the type, prevention, etiology, and pathogenesis of stroke; the concept of stroke rehabilitation; the importance of early rehabilitation; best practices in stroke rehabilitation; and nursing care.

### Cognitive training

All the patients will receive CT based on the 2010 National Stroke Foundation’s *Clinical Guidelines for Stroke Management* [34]. The intervention group will receive CT and acupuncture therapy simultaneously. The therapists will tailor the treatment in accordance with each patient’s assessment results, including different aspects of CT (attention, memory, orientation, visual spatial ability, execution, or calculation and so on). Furthermore, the level of difficulty will be based on patient’s progress. The period of treatment will be sustainable for 12 weeks, at 30 minutes per day for five days a week.

### Acupuncture therapy

The intervention group will be treated on two acupuncture points (Baihui (DU20) and Shenting (DU24)). The acupoint-location method is based on bone proportional cun. Bone proportional cun is mainly marked as the primary landmarks by using joints on the surface of the body; the length between the two joints will be discounted for a certain amount of discretion in order to determine the location of the acupoints. Baihui (DU20) is located on the head, 5 B-cun superior to the anterior hairline, on the anterior median line; or when the ears are folded, Baihui (DU20) is located at the midpoint of the connecting line between the auricular apices. Shenting (DU24) is located on the head, 0.5 B-cun superior to the anterior hairline, on the anterior median line.

Acupuncture will be performed by acupuncturists who are TCM licensed, and have at least three years of experience. The acupuncture procedure starts with the patient in the sedentary or supine position. After acupoint area sterilization, the acupuncturist’s left hand should fix the skin of the stimulus area, and the right hand should hold the disposable sterile acupuncture needle (Huatuo brand, model 0.30 mm × 25 mm, Suzhou Medical Appliance Factory, China). Then, the needle is inserted into the acupoints subgaleally from the front to the rear along the scalp, at an angle of 10 to 20 °, to a depth of approximately 25 to 35 mm. Following insertion, stimulation of the acupuncture point is applied until the *de qi* sensation is achieved, which means that patients experience a certain feeling with a mixture of sourness, numbness, distension, and pain from the acupuncture. The acupuncturist will use the manipulation technique with rapid twirling performed for two to three minutes every 10 minutes, at a frequency of 180 to 300 times/min. Meanwhile, CT will be performed constantly for 30 minutes throughout the acupuncture therapy. Following the treatment session, the needles will be withdrawn, and a dry sterilized cotton ball will be slightly pressed the holes of needles to avoid bleeding. The treatment will be given once a day for five times a week, lasting for 12 continuous weeks.

### Follow-up

After the 12-week treatment period, there is an outpatient 24-week follow-up period. The medical staff will follow up on the participants via telephone. Each participant is contacted once every four weeks, keeping record of the medications and rehabilitation therapies. On the final week of the follow-up period (24 weeks post-intervention), participants will be referred for clinical evaluation in order to assess their functional status, including cognitive function, quality of life, and ADL.

### Outcome measures

In this study, all the primary and secondary outcomes measures will be tested at baseline (−2 to −1 week) and after the intervention period (12 weeks). After follow-up (36 weeks), the cognitive function, quality of life, and ADL outcomes (MMSE, MoCA, SF-36, and MBI) will also be evaluated. All outcome assessments will be independently performed by experienced and blinded assessors. A summary of all the measures in the trial is shown in Table 1.

### Primary outcomes

#### Mini-Mental State Examination

The Mini-Mental State Examination (MMSE), was first introduced by Folstein *et al*. in 1975 [35], and we will use the revised Chinese MMSE version [36]. It is a brief 30-point questionnaire, divided into 10 items: time and place orientation, short-term memory, attention and calculation, recall, language skills, repetition, complex commands, reading, writing, and copying of pentagons. When the resulting MMSE is 24 points or less, this means that the patient may suffer from some degree of cognitive impairment. The total score of the individual items demonstrates the recent severity of cognitive impairment.

### Secondary outcomes

#### Montreal Cognitive Assessment

The Montreal Cognitive Assessment (MoCA) [37] is a 30-point test that covers eight cognitive domains, including: visual spatial ability and execution, naming, memory, attention, language, abstraction, delayed recall, and orientation. Given the MMSE’s limitation in detecting early dementia [38], the MoCA evaluates participants who suffer from cognitive complaints. The revised Chinese MoCA version will be used in this trial.

#### Test of Attentional Performance

The Test of Attentional Performance (TAP, Version 2.3) [39], a computer-aided standard neuropsychological test, is used to assess attention disorders. Four of its sub-tests were chosen for this trial: alertness for reaction, general anticipation (which belongs to general attention); Go/No Go, and incompatibility for selective attention and divided attention, which is essential in every attention assessment. The TAP measures reaction time, and the number of errors and omissions. The available TAP in Chinese will be used in this study.

#### Wechsler Memory Scale-Revised in Chinese

Memory will be evaluated using the Wechsler Memory Scale (WMS). The Wechsler Memory Scale-Revised in Chinese (WMS-RC) will be used [40]. The test consists of three sub-functions (11 sub-tests): long-term memory (including information and orientation, forward recite digit (1 to 100), backward recite digit (100 to 1), and accumulation), short-term memory (including visual recognition, picture recall, visual reproduction, associative learning, touch, and comprehension memory), and immediate memory (including digit span). The results of the WMS-RC are expressed by memory quotient (MQ). We will collect MQ values and all sub-test values for analysis.

#### The Trail Making Test and the Stroop test

Executive function will be measured with the TMT and the Stroop test. The TMT is one of the most popular neuropsychological tests and provides information on visual search and executive functions [41]. It consists of part A and part B (TMT-A and TMT-B). In part A, the participants are asked to make lines from 1 to 25, while in part B, subjects should make lines from 1 to 25 while alternating between circles and squares [42]. This study also adopts the Color-Word Stroop interference test using E-prime to assess subjects’ executive function. Subjects were asked to judge if the color of the top row in the computer screen corresponds to the color name written at the bottom row, and press the left or the right button, respectively [43].

#### Fugl-Meyer Motor Assessment

The Fugl-Meyer Motor Assessment evaluates upper and lower limb motor functions systematically [44]. The maximum score is 100, the upper extremity score is 66, and the lower extremity score is 34 [45].

#### Modified Barthel Index

ADL will be evaluated by the MBI [46]. The Barthel Index was first introduced in 1965 and consists of 10 items: feeding, personal hygiene, dressing, bathing, toileting, bowel control, bladder control, chair-bed transfer, ambulation or wheelchair, and stair climbing [47]. The BI was modified in 1989 to improve content reliability and internal consistency, resulting in the MBI [48]. Each item’s score ranges from 0 to a maximum that varies from 5 to 15. The score indicates the ADL ability range from inability to independence. Higher scores suggest better functional and independent ADL abilities, thus a score of 100 means total independence.

#### 36-Item Short Form Health Survey

Health-related quality of life is measured using the Chinese version of the 36-item Short Form Health Survey [49]. This questionnaire consists of 36 items related to physical and mental health, which are grouped in eight dimensions: physical functioning; role-physical, or the limitation in daily role functioning due to physical problems; role-emotional, or the limitation in daily role functioning due to emotional problems; bodily pain; general health perception; vitality; social functioning; and mental health perception, relating to physical and mental health respectively [50].

#### Functional Magnetic Resonance Imaging

Resting-state functional magnetic resonance imaging (fMRI) will be used in this study to examine longitudinal changes for cognitive impairment patients after stroke, and to investigate the relationship between imaging changes and cognitive recovery [51]. Subjects will be scanned twice (before and after treatment), using a 3.0 T signal MRI scanner (GE Healthcare, Little Chalfont, UK) with a birdcage head coil [52].

#### Event-related potentials

Cognitive function will be measured by event-related potentials (ERPs). ERP P300 is related to cognitive function closely, which can reflect information processing speeds, attention, and the updating of working memory [53]. Reaction time and correctness rates (correctly pressing the button for target and non-target stimulus presentations) will be measured to quantify the cognitive function, using the software system of Neuroscan ERPs (SynAmps^2^ Model 8050, Compumedics USA, Ltd, Charlotte, USA)

### Safety assessments

Whenever an adverse event (AE) occurs during the treatment or follow-up period, the medical staff will manage it accordingly, while researchers will document any AE in detail. Every participant will have the right to report AEs at any given point in time during this trial, and will receive appropriate medical attention. Serious AEs will be immediately reported to the primary investigator and the Human Research Ethics Committee of FJTCM to determine whether the participant needs to be withdrawn from the study.

### Economic evaluation

Cost-effectiveness and incremental cost-effectiveness (the required cost of improvement in cognitive function) ratios, will be the primary economic outcomes in this trial [54]. The cost of interventions comprises direct costs and indirect costs. Any expenses involving rehabilitation treatments, rehabilitation assessment fees, auxiliary equipment, and other direct medical expenses associated with stroke or direct non-medical cost (including rehabilitation related transportation, special diets, nurse escort fee, and other costs related directly to the medical expenses) throughout the rehabilitation period are classified as direct costs. In addition, the number of workdays lost by participants during the treatment and follow-up period will be used to evaluate indirect costs. Incremental quality adjusted life years will be the total of difference in duration of rehabilitation effects as weighted by cognitive function between intervention group and control group during the treatment and follow-up period.

### Data collection and management

Firstly, screeners are responsible for the acquisition of demographic and baseline characteristic data when the participants are recruited. Outcome assessors play an important role in evaluating the primary and secondary outcomes at baseline, 12 weeks (end of intervention), and 36 weeks (after the 24-week follow-up period). In each center, after the source data has been added to the case report form, two research assistants who are not engaged in either treatment or evaluation will enter those source data into an electronic data capture database through the scientific research management platform of FJTCM. Only the project manager has the right to log into every center’s database. Moreover, a statistical manager will be in charge of initial data organizing, identifying, coding, and converting data to make sure the format is proper for data analysis. Meanwhile, double data entry will be used to promote data quality. All materials related to the participants’ privacy will be kept confidential by the way of coding. Besides, all study documents will be retained securely for five years after completion of the study, and the result of our study will be published in open access journals.

Moreover, this trial has been monitored for safety control by an independent Data and Safety Monitoring Board (DSMB), consisting of two senior therapists and one rehabilitation physician who are experienced in post-stroke cognitive impairment and have no direct involvement in the study. The DSMB is an independent board and is responsible for source data validation, and AE management and reporting. For safety concerns, the DSMB may have the right to recommend termination or modification of the research, according to their clinical judgment.

### Statistical methods

All data will be analyzed using SAS version 8.2 (SAS institute Inc., Cary, NC, USA) by an independent blinded statistician. A statistical significance level of 0.05 will be used. For statistical description, continuous variables will be presented for each group using the mean ± standard deviation for normal distribution, and median and interquartile range for non-normal distribution. Categorical variables will be presented by using proportions with their standard error. In order to compare the primary and secondary outcomes between groups, measured at baseline, post-intervention and post-follow-up, the t-test or Mann–Whitney U test will be used for continuous variables and the Pearson chi-squared or Fisher’s exact test will be used for categorical variables. Non-parametric tests, Pearson chi-squared test, or Fisher’s exact test will be used for expressing changes in effect sizes of primary and secondary outcomes between groups before and after treatment, and during the follow-up period (at 12 weeks and 24 weeks).

To adjust the confounding influence if necessary, a general linear model or linear regression will be applied for dependent continuous variables, and a logistic regression model for dependent categorical variables. The analysis of the primary outcome will be expressed by using full analysis set with the principle of intention-to treat and the per-protocol set. The analysis of the secondary outcomes will be performed using the per-protocol set. The analysis of AE will be undertaken by using a chi-square test or Fisher’s exact test. Lastly, the economic evaluation will be assessed using cost-effectiveness analysis. Missing data will be imputed by the last observation carried forward rules.

## Discussion

Post-stroke cognitive impairment is highly prevalent all over the world [16]. It significantly impairs patients’ participation in rehabilitation, engagement in daily activities, and their quality of life [55]. Meanwhile, the high economic burden that stroke rehabilitation imposes on family and society is growing [56]. Moreover current therapies, mainly CT and pharmacological treatment, lack sufficient evidence to support their effectiveness due to the complexity of cognitive impairment management [6]. The recent use of combined therapy has increasingly attracted attention, offering a new prospect in PSCI treatment [17].

Acupuncture is considered to be effective in improving cognition and has the advantages of being simple, convenient, efficient, and inexpensive, without severe adverse effects [20, 57]. A systematic review [58] has proposed that acupuncture improves cognitive function impairment in patients with PSCI, but the lack of high quality research has prevented the development of evidence-based recommendations for clinical practice. This review also recommends that future clinical trials should focus on the effect of acupuncture treatments combined with a second treatment, aiming to find the most cost-effective therapy combination that promotes the greatest improvement in stroke rehabilitation.

Previous studies have been limited by random methodology and small sample sizes [26, 27]. Thus, a prospective, multicenter, large sample, single-blinded, randomized controlled trial designed under strict systematic operational and high quality practices is needed to evaluate whether a therapeutic regimen of TCM rehabilitation leads to a more effective improvement in PSCI.

A notable difference between our study and former studies is that this trial will investigate, some domain-specific cognitive functions as well as general cognitive state. According to one article, patients with PSCI will mainly exhibit dysfunctions in attention, memory, and executive function [6]. To evaluate domain-specific cognitive function various measures were selected: the TAP to assess attention, the WMS-RC to evaluate memory, and the TMT and Stroop test to measure executive function. In this study, detailed information on performance in multifarious tasks designed for CT will be recorded, allowing an insightful outcome analysis by taking into account the severity of and inter-relationships among the cognitive deficits in various domains. Besides, fMRI and ERPs will be included in this trial as objective indicators of cognitive function [59–61]. Since many stroke patients who suffer from PSCI and movement disorders cannot understand and/or perform tasks adequately, resting-state fMRI was chosen over tasking-state fMRI for this study [62]. Meanwhile, ERP P300 and resting-state fMRI make it possible to provide a potential mechanism for the therapeutic effects of this trial’s approach, and to further explore the relationship between cognitive functional networks and brain structure, thus allowing the researchers to investigate the relationship between subjective scales and objective indicators.

Adhere to the SPIRIT guidelines, a prospective, multicenter, large sample, randomized controlled trial with blinded assessments has been designed [63]. This protocol design has several strengths: (1) it is a multicenter trial that will be carried out in seven inpatient and outpatient stroke rehabilitation units, which enhances the study’s external validity and sample representativeness; (2) to guarantee quality, all staff in the study are required to complete training on standard operating procedures of the study protocol before recruiting; (3) this study is centrally randomized to guarantee adequate allocation concealment and blinded assessment of results [64]; (4) a 12-week intervention and 24-week follow-up assessment can provide reliable long-term effect evidence for a therapeutic regimen of TCM rehabilitation treatment and its health economics in PSCI clinical practice.

However, this protocol design also has potential limitations: for example, it is not a double-blinded, placebo-controlled trial. From the perspective of evidence-based medicine, randomized, double-blind, placebo-controlled trials are the best the trials that render the highest evidence quality, but their feasibility is a major difficulty in non-pharmacological research areas [65]. Considering the ethics and participants’ adherence, blinding seems difficult for acupuncture in a clinical study, thus randomized, double-blinded, placebo-controlled trials may be impossible. Considering these facts, this trial attempts to reduce possible bias through blinding both outcome assessors and data analyst. Further, potential spontaneous recovery at the early stage of stroke may have the confounding effects on the trial.

To conclude, this trial aims to evaluate, under strictly systemized operational and high quality practices, whether a therapeutic regimen of TCM rehabilitation can effectively improve cognitive functions recovery after stroke under strict standard operational practices. If this trial is successfully carried out and renders high quality evidence to support a therapeutic regimen of TCM rehabilitation for PSCI management, this low cost and feasible therapy could be implemented on a larger scale in clinical and community settings.

## Trial status

Participant enrollment started in July 2014. The trial is expected to be completed by the end of December 2016.

## Additional file

Additional file 1:
**The names of seven ethical bodies**

## Abbreviations

ADL: Activities of daily living
AE: Adverse event
CT: Cognitive training
DSMB: Data and Safety Monitoring Board
ERP: Event-related potentials
FJTCM: Fujian University of Traditional Chinese Medicine
fMRI: Functional magnetic resonance imaging
MBI: Modified Barthel Index
MMSE: Mini-Mental State Examination
MoCA: Montreal Cognitive Assessment
PSCI: Post-stroke cognitive impairment
SF-36: 36-Item Short Form Health Survey
TAP: Test of attentional performance
TCM: Traditional Chinese Medicine
TMT: Trail Making Test A and B
WMS-RC: Wechsler memory scale-revised in Chinese
