# Supplementary material for: The effect of a therapeutic regimen of Traditional Chinese Medicine rehabilitation for post-stroke cognitive impairment: study protocol for a randomized controlled trial
Source: Trials. 2015 Jun 16;16:272. doi: 10.1186/s13063-015-0795-x (PMC4485558; doi:10.1186/s13063-015-0795-x)
Supplement: Additional file 1: — The names of seven ethical bodies [file 13063_2015_795_MOESM1_ESM.doc]

**Additional file 1 The names of seven ethical bodies**

| Number | The names of ethical bodies | Approval No. |
| --- | --- | --- |
| 1 | Fujian University of Traditional Chinese Medicine Subsidiary Rehabilitation Hospital | 2013KY-005-01 |
| 2 | Traditional Chinese Medicine Hospital of Xinjiang Uygur Autonomous Region | 2013XE005 |
| 3 | Guangdong Province Traditional Chinese Medical Hospital | B2014-020-01 |
| 4 | The first affiliated hospital of Jinan university | (2014) Ethical review approval documents 25th |
| 5 | The affiliated Huashan Hospital of Fudan University | (2014) Interim review 262th |
| 6 | Zhongshan Hospital of Traditional Chinese Medicine | 2014ZSZY-LLK-002 |
| 7 | ShiyanTaihe Hospital | (2014001) Research review meeting 2nd |
